# The *JAK2* V617F mutation in retinal vein or artery occlusion

**Published:** 2019-03-04

**Authors:** Stephen E. Langabeer

**Affiliations:** 1 Cancer Molecular Diagnostics, St. James's Hospital, Dublin, Ireland

## ⁯⁯⁯⁯

**Dear Editor,**

The *JAK2* V617F mutation is the most commonly acquired driver mutation of the myeloproliferative neoplasms (MPN), detected in more than 95 % of patients with polycythaemia vera and in 50-60 % of patients with essential thrombocythaemia and primary myelofibrosis (Ferreira Cristina et al., 2018[4]). Patients with these MPN have a considerably increased risk of thrombosis, particularly at uncommon sites (Ball et al., 2018[2]). Risk factors for retinal vein/artery occlusion include atherosclerosis, inflammation and compression, with thrombosis possible due to a malignancy-associated thrombophilic state (Ip and Hendrick, 2018[5]). Retinal vein/artery occlusion is recognised as a rare but recurrent presenting feature of MPN (Tache et al., 2005[7]; Arikan et al., 2011[1]; Dhrami-Gavazi et al., 2015[3]; Rao et al., 2016[6]). 

In order to address the clinical value and laboratory impact of requesting *JAK2* V617F mutation status in patients with retinal vein/artery occlusion, a retrospective audit was performed on *JAK2* V617F requests received at a molecular diagnostics centre for haematological malignancies. From January 2006 to September 2018 inclusive, 17332 diagnostic requests for *JAK2* V617F mutation analysis were received. Of these, 29 requests (0.2 %) were identified that included clinical details provided of either retinal vein/artery occlusion (n=11) or thrombosis (n=18). The median age was 49 years and comprised 12 males and 17 females. MPN-associated haematological abnormalities were noted as either erythrocytosis (n=4), raised haemoglobin and/or haematocrit (n=6), thrombocytosis (n=9) or not provided (n=10). Using a standardised screening assay unchanged throughout the audit period, the *JAK2* V617F mutation was detected in five patients (17.2 %) with either raised haemoglobin and/or haematocrit (n=3) or thrombocytosis (n=2).

While the number of requests in patients with a retinal vein/artery occlusion does not appreciably impact on overall laboratory workload, reflexive screening for the *JAK2* V617F, particularly in those patients with the aforementioned haematological abnormalities, is justified in order to identify an underlying MPN.

## Conflict of interest

The author declares no conflict of interest.
